# Spatial and Temporal Analysis of Severe Fever with Thrombocytopenia Syndrome in Anhui Province from 2011 to 2023

**DOI:** 10.1007/s44197-024-00235-3

**Published:** 2024-09-02

**Authors:** Xiu-Jie Chu, Dan-Dan Song, Na Chu, Jia-Bing Wu, Xiaomin Wu, Xiu-Zhi Chen, Ming Li, Qing Li, Qingqing Chen, Yong Sun, Lei Gong

**Affiliations:** 1 Department of Acute Infectious Disease Prevention and Control, Anhui Provincial Center for Disease Control and Prevention, Hefei, Anhui China; 2 Microbiological Laboratory, Anhui Provincial Center for Disease Control and Prevention, Hefei, China; 3 Microbiological Laboratory, Public Health Research Institute of Anhui Province, Hefei, China

**Keywords:** Severe fever with thrombocytopenia syndrome, Spatiotemporal clustering, Spatial autocorrelation, Spatiotemporal scanning

## Abstract

**Objective:**

To analyze the spatial autocorrelation and spatiotemporal clustering characteristics of severe fever with thrombocytopenia syndrome(SFTS) in Anhui Province from 2011 to 2023.

**Methods:**

Data of SFTS in Anhui Province from 2011 to 2023 were collected. Spatial autocorrelation analysis was conducted using GeoDa software, while spatiotemporal scanning was performed using SaTScan 10.0.1 software to identify significant spatiotemporal clusters of SFTS.

**Results:**

From 2011 to 2023, 5720 SFTS cases were reported in Anhui Province, with an average annual incidence rate of 0.7131/100,000. The incidence of SFTS in Anhui Province reached its peak mainly from April to May, with a small peak in October. The spatial autocorrelation results showed that from 2011 to 2023, there was a spatial positive correlation(*P* < 0.05) in the incidence of SFTS in all counties and districts of Anhui Province. Local autocorrelation high-high clustering areas are mainly located in the south of the Huaihe River. The spatiotemporal scanning results show three main clusters of SFTS in recent years: the first cluster located in the lower reaches of the Yangtze River, the eastern region of Anhui Province; the second cluster primarily focused on the region of the Dabie Mountain range, while the third cluster primarily focused on the region of the Huang Mountain range.

**Conclusions:**

The incidence of SFTS in Anhui Province in 2011–2023 was spatially clustered.

## Introduction

In recent years, the global prevalence of emerging infectious diseases such as COVID-19 and monkeypox has posed significant challenges to public health and necessitated enhanced surveillance measures for monitoring emerging infectious disease outbreaks. Severe fever with thrombocytopenia syndrome(SFTS) is an emerging infectious disease that was initially identified in rural areas of Henan province, China, in 2009 and has rapidly spread to other provinces in central, eastern, and northeastern regions [1]. Subsequently, it has been reported in Japan, South Korea, Vietnam, and other countries and regions [2, 3]. SFTS is caused by severe fever with thrombocytopenia syndrome virus(SFTSV). Thrombocytopenia, leukopenia, fever, and gastrointestinal symptoms are common manifestations of SFTS [4]. In some cases, the disease can lead to death due to shock, multi-organ failure, and other complications [4]. Currently, the average case fatality rate of this disease stands at 16.2% [5]. The impact of SFTS on people’s lives and health as well as economic development has been significant in the central and eastern regions of China.

Previous studies have demonstrated the spatial aggregation of SFTS incidence, with a majority of patients being farmers residing in mountainous, forested, and hilly areas [6, 7]. Anhui belongs to one of four clusters of SFTS incidence in China known as the Huaiyangshan cluster which includes Henan, Hubei, Jiangsu, and Zhejiang provinces [8]. In recent years, there has been a clear increasing trend in SFTS cases reported in Anhui Province making it one of highest affected provinces nationwide in 2023. Studies have indicated that tick bites are considered as main route for transmission of SFTSV, particularly through *Haemaphysalis longicornis*(*H. longicornis*) ticks; most patients initially diagnosed with SFTS had a history of tick bites before onset[9, 10]. The distribution pattern of *H. longicornis* closely correlates with meteorological and geographical factors exhibiting significant seasonal characteristics [9]. Migratory birds carrying SFTSV-infected ticks may pose potential risks for transregional transmission [11]. Furthermore, Zhejiang and Jiangsu provinces adjacent to Anhui within the Huaiyangshan cluster also exhibit evident spatiotemporal clustering patterns regarding SFTS incidence [12–14]. However, current research lacks specific analysis for the persistently high number of SFTS cases observed in Anhui province, and no corresponding study on spatio-temporal clustering patterns related to SFTS has been conducted yet.

Given the severe impact of SFTS on both public health and the socio-economic landscape, it is imperative to employ geographic information systems to comprehend the epidemic characteristics of SFTS prevalence areas. Gaining insight into the epidemic profile plays a pivotal role in studying disease progression and monitoring outbreaks. Furthermore, identifying specific regions with high incidence rates can aid in further elucidating causative factors and take targeted prevention and control measures. Therefore, we utilized geographic information systems to analyze spatial autocorrelation and spatiotemporal clustering patterns of fever with platelet syndrome in Anhui Province from 2011 to 2023, providing a scientific foundation for fever with platelet syndrome prevention and control.

## Methods

### Case Definition

When patients meet the following criteria patients are defined as suspected cases. (1) Acute fever (≥ 38.0℃) accompanied by symptoms such as fatigue and nausea. (2) Epidemiological risk factors including work and travel history in relevant risk areas or exposure to ticks within two weeks before onset. (3) Patients presenting with thrombocytopenia and leukopenia.

A suspected case is considered confirmed if it meets one or more of the following criteria: (1) Detection of positive SFTSV nucleic acid. (2) Serum sample showing a four-fold higher titer of SFTSV IgG antibody in the recovery phase compared to the acute phase. (3) Isolation of SFTSV pathogen in cell culture.

### Data Source

Data of SFTS cases were collected from the Chinese Center for Disease Control and Prevention of infectious disease network report surveillance system. The SFTS cases from January 2011 to December 2023 were counted by address and date of onset. The cases referred to in this paper included clinically diagnosed cases and laboratory-confirmed cases. The population data of each county is from the Anhui Census Yearbook − 2020.

### Spatial Autocorrelation

Spatial autocorrelation analysis was conducted using GeoDa 1.18 software [15] to examine the incidence of SFTS in Anhui Province from January 2011 to December 2023, with the county(district) as the basic unit. The primary analysis focused on the spatial distribution of SFTS cases, encompassing both global and local autocorrelation analyses. Global autocorrelation was employed to explore the overall spatial pattern within the study area, utilizing Moran’s I index as the main indicator. Moran’s I ranges from − 1 to 1, where values greater than 0 indicate positive spatial correlation and closer proximity to 1 signifies stronger spatial aggregation. Conversely, negative values approaching − 1 suggest significant spatial negative correlation. A value of zero for Moran’s I indicate no spatial autocorrelation exists. To assess its significance, a Z-test was performed at a significance level(α) of 0.05.

Furthermore, considering the presence of spatial heterogeneity, it is plausible that the distribution of SFTS may exhibit temporal and spatial variations across different counties. Hence, it becomes imperative to employ local indicators capable of detecting local spatial autocorrelation to ascertain the degree of spatial autocorrelation between various regions within the study area. To visualize these localized patterns, we utilized an aggregation map known as Local Indicators of Spatial Association(LISA), based on region-specific incidence rates derived from our analysis results. We classified regions exhibiting statistically significant clustering into four distinct types: high-high clustering, high-low clustering, low-high clustering, and low-low clustering. The criterion for identifying an area as a cluster is that it must maintain this classification for six years.

### Spatio-Temporal Scan

SaTScan10.0.1 software [16] was utilized to identify and assess the temporal and spatial aggregation areas of SFTS. SaTScan is a freely available software that employs scan statistics to detect regions with excessive risk in time, space, or space-time, while generating P-values for these regions through Monte Carlo hypothesis testing. SaTScan examines both time and/or space to investigate potential clusters by comparing observed incidence rates with expected rates assuming random distribution within each location window. The SaTScan software incorporates both temporal and spatial dimensions, employing dynamically evolving cylindrical shapes to scan various spatiotemporal windows. The cluster with the highest Log-Likelihood Ratio (LLR) is identified as the most probable cluster [17]. In this study, we employed a discrete Poisson distribution to construct a probability model and adjusted maximum spatial cluster size to 10% of population at risk within spatial windows along with maximum time cluster size set at 20% of study period duration. Within each time period, primary clusters are defined as regions exhibiting maximum log-likelihood ratio since they have highest likelihood of aggregation occurring there. Other statistically significant clusters are designated as secondary or tertiary clusters.

### Statistical Analysis

If the data conforms to normal distribution, the mean and standard deviation (x ± s) will be used; otherwise, the median of the quartile range(M[Q1, Q3]) will be used. The counting data is described in terms of frequency and scale. We conducted a matching process between the home address administrative code of SFTS cases and the map code of each county to establish a QGIS database for SFTS. Additionally, we utilized QGIS 3.30 software [18] to visualize the results of spatial autocorrelation analysis and spatio temporal scanning analysis.

## Results

### Descriptive Results

During the period from 2011 to 2023, Anhui Province reported a total of 5720 cases of SFTS, with an average incidence rate of 0.7131 per 100,000 population. Among these cases, there were 183 deaths resulting in a fatality rate of 3.20%. See Table 1.

**Table 1:** The morbidity and mortality condition in Anhui Province from 2011 to 2023

Year	Report Cases	Incidence rate(/100,000)	Fatal Cases	Incidence rate(/100,000)	Fatality rate(%)
2011	61	0.1025	10	0.0168	16.39
2012	59	0.0972	4	0.005	6.78
2013	81	0.1353	5	0.01	6.17
2014	230	0.3814	5	0.0083	2.17
2015	258	0.4176	10	0.0164	3.88
2016	474	0.7715	9	0.0146	1.9
2017	338	0.5456	8	0.0129	2.37
2018	330	0.5276	12	0.0192	3.64
2019	442	0.699	16	0.0253	3.62
2020	641	1.0069	39	0.0613	6.08
2021	544	0.8914	11	0.018	2.02
2022	840	1.3741	22	0.036	2.62
2023	1422	2.3207	32	0.0522	1.89

**Fig. 1:**
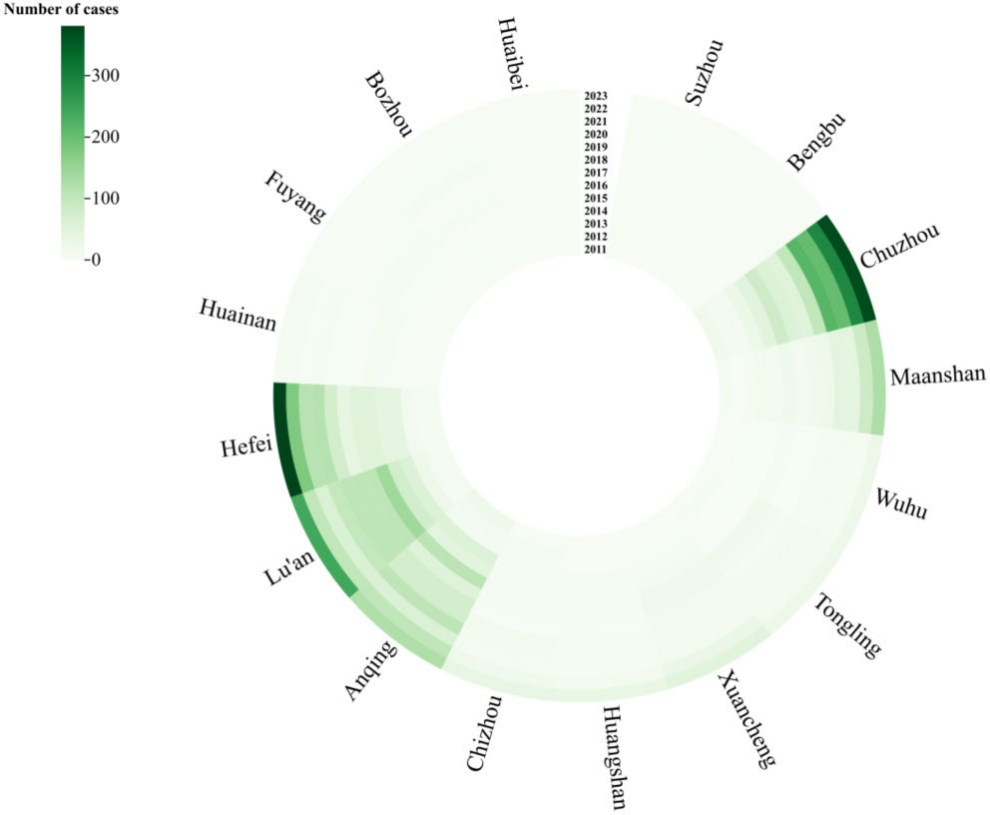
Time distribution of SFTS in cities of Anhui Province from 2011 to 2023

Among the 16 cities in Anhui Province, Chuzhou City, Lu’an City, Hefei City, Anqing City, and Maanshan City exhibited the highest number of SFTS with 1,497, 1,140, 1,117, 859 and 420 cases respectively. These five cities accounted for a majority proportion(87.99%) of the total incidence. Furthermore, there has been an upward trend in the number of cases reported in these top five cities over recent years. as depicted in Fig. 1.

**Fig. 2:**
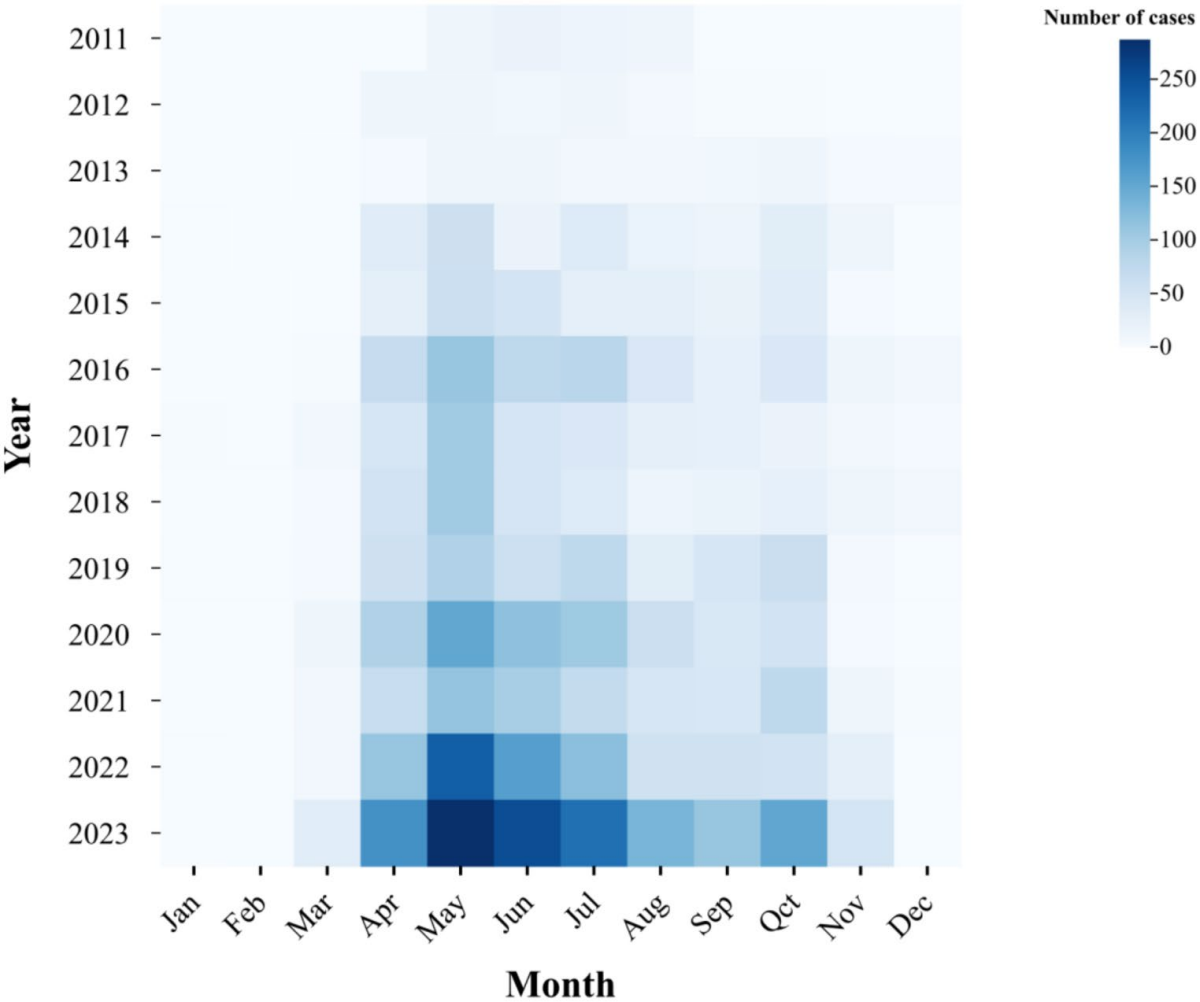
The monthly distribution chart in Anhui Province from 2011 to 2023

In terms of seasonal patterns, the incidence of SFTS in Anhui Province exhibits a prominent peak during April and May, accompanied by a minor peak in October. See Fig. 2.

### Spatial Autocorrelation Analysis

#### Global Spatial Autocorrelation Analysis

The global spatial autocorrelation analysis displayed the results of Moran’s I, indicating a significant clustering pattern in the regional distribution of SFTS cases in Anhui Province from 2011 to 2023. The annual Moran’s I values were as follows: 0.272, 0.195, 0.116, 0.228, 0.378, 0.270, 0.289, 0.348, 0.296, 0.503, and finally stabilized at around 0.454 − 0.364 (Table 2).

**Table 2:** Global spatial autocorrelation analysis results of fever with thrombocytopenia syndrome in Anhui Province from 2011 to 2023

Year	Moran′ I	Z value	*P* value
2011	0.272	4.777	0.001
2012	0.195	3.234	0.006
2013	0.116	2.116	0.035
2014	0.228	4.418	0.001
2015	0.378	6.482	0.001
2016	0.270	5.417	0.001
2017	0.289	5.444	0.001
2018	0.348	6.349	0.003
2019	0.296	5.056	0.002
2020	0.503	8.485	0.001
2021	0.454	7.679	0.001
2022	0.414	7.034	0.001
2023	0.364	5.911	0.001

#### Local Autocorrelation Results

The local autocorrelation analysis revealed an increasing trend in the high-high aggregation and low-low aggregation areas of SFTS in the province from 2011 to 2023. The high-high aggregation areas primarily encompass Lai’an County, Langya District, Nanqiao District, Quanjiao County, Qianshan City, Lujiang County, Yuexi County, Shucheng County, and Huoshan County. These areas consist of four counties and districts in Chuzhou City, two counties and districts in Anqing City and Lu’an City respectively, as well as one county and district in Hefei City. Notably, new

**Fig. 3:**
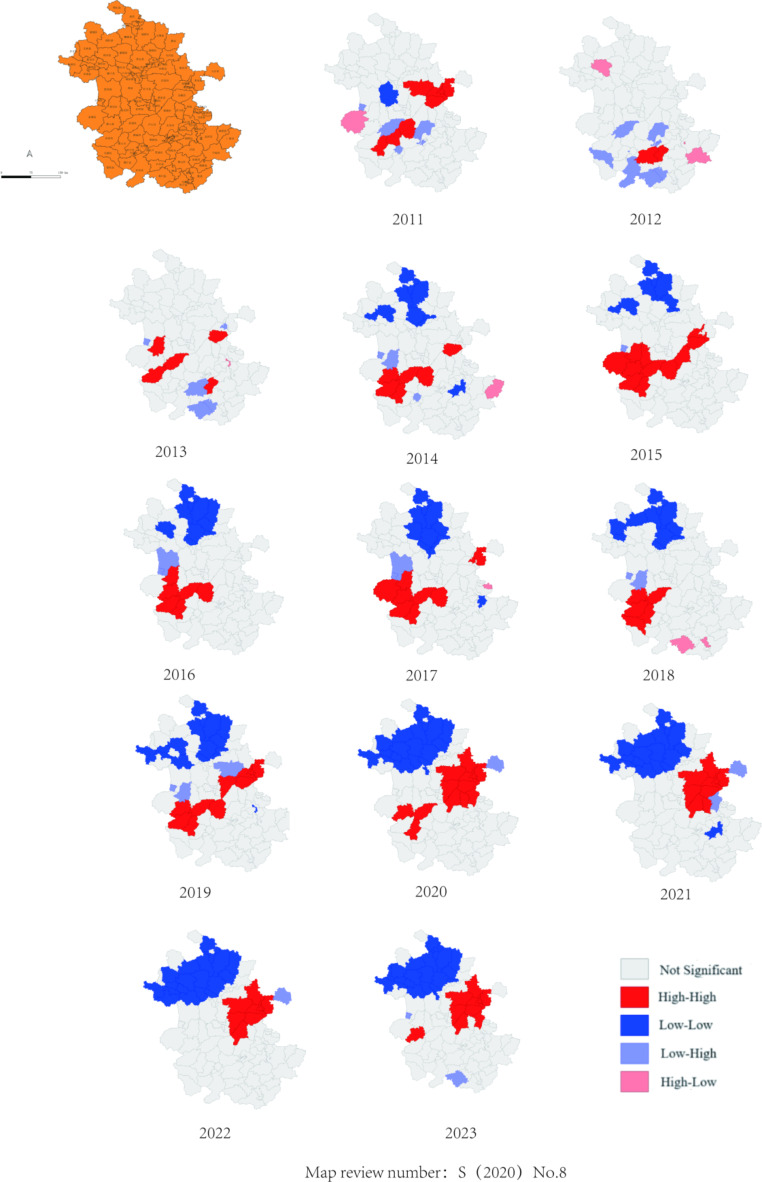
Local spatial autocorrelation analysis of severe fever with thrombocytopenia syndrome in Anhui Province from 2011 to 2023

High-high aggregation areas have emerged recently. For example, Feidong County has consistently been a high-high aggregation area from 2019 to 2023, however it was relatively stable before 2019. The low-low accumulation area mainly comprises Xiao County, Lieshan District, Yongqiao District, Suixi County, Huaiyuan County, Huaishang District, Yingquan District, Lixin County, Guzhen County, Panji District. It primarily includes three counties in Bengbu City along with two counties each from Suzhou City and Huaibei City respectively; additionally one county each from Bozhou City, Huainan City and Fuyang City is also part of this category(see Fig. 3).

### Spatiotemporal Clustering Analysis

**Fig. 4:**
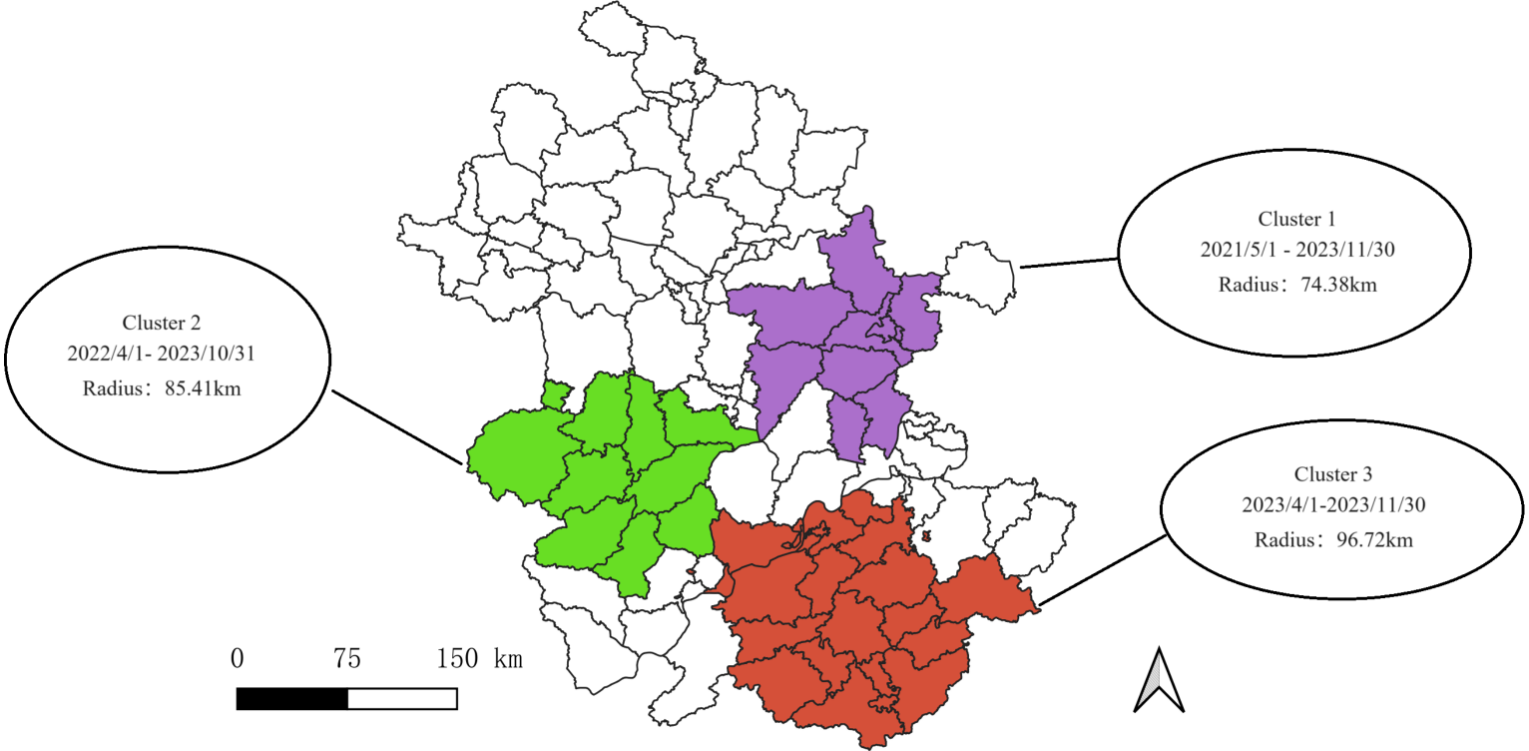
Spatio-temporal scanning results of SFTS in Anhui Province from 2011 to 2023

SatScan results showed that the incidence of SFTS was clustered, the whole Province was divided into three major clusters of SFTS from 2011 to 2023. Among them, the first cluster area covered 9 counties and districts, including Dingyuan County, Feidong County, Hanshan County, Hexian County, Laian County, Langya District, Mingguang City, Nanqiao District and Quanjiao District were gathered from May 1, 2021 to November 30, 2023. The area radius was 74.38 km, the relative risk was 17.19, and the LLR was 2063.28(*P* < 0.001). The second aggregation area covered 10 counties, including Feixi County, Huoshan County, Jinan District, Jinzhai County, Qianshan County, Shucheng County, Tongcheng County, Yeji District, Yuan District and Yuexi County. The aggregation time was from April 1, 2022 to October 31, 2023. The area radius was 85.41 km, the relative risk was 8.97, and LLR was 689.44(*P* < 0.001). The third gathering area covers 21 counties and regions, including Zongyang County, Fanchang County, Guichi County, Huangshan District, Huizhou District, Jixi County, Jingxian County, Jingde County, Nanling County, Ningguo City, Qimen County, Qingyang County, Shexian County, Shitai County, Tongguan District, Tunxi District, Xiuning County, Yixian County, Yian District, Yingjiang District and Jiaoqu District. The gathering time is from April 1, 2023 to November 30, 2023. The area radius is 96.72 km, and the relative risk is 5.07. The LLR was 106.6(*P* < 0.001). (See Table 3; Fig. 4). It is worth noting that most of the high-high clustering regions in spatial autocorrelation are also clustering regions obtained by spatiotemporal scanning (8/9).

**Table 3:** The results of spatial-temporal scanning

Clusters	Clusters 1	Clusters 2	Clusters 3
Coordinates	32.315421 N, 118.182134 E	31.283755 N, 116.234860 E	30.254484 N, 118.091376 E
Radius	74.38 km	85.41 km	96.72
Time frame	2021/5/1–2023/11/30	2022/4/1 to 2023/10/31	2023/4/1 to 2023/11/30
Location counties	Dingyuan, Feidong, Hanshan, Hexian, Laian, Langya, Mingguang, Nanqiao, Quanjiao	Feixi, Huoshan, Jinan, Jinzhai, Qianshan, Shucheng, Tongcheng, Yeji, Yu’an, Yuexi	Zongyang, Fanchang, Guichi, Huangshan, Huizhou, Jixi, Jingxian, Jingde, Nanling, Ningguo, Qimen, Qingyang, Shexian, Shitai, Tongguan, Tunxi, Xiuning, Yixian, Yian, Yingjiang, Jiaoqu
Population	4,383,619	5,774,912	5,427,023
Number of cases	1146	545	131
Expected cases	82.20	66.42	26.30
Relative risk	17.19	8.97	5.07
LLR	2063.28	689.44	106.6
P value	< 0.001	< 0.001	< 0.001

## Discussion

Since the first isolation of a novel Bunyavirus in China in 2011, the annual incidence of SFTS in our province has consistently shown an upward trend, aligning with the national development tendency of SFTS. The average incidence rate of SFTS in Anhui Province from 2011 to 2023 reached 0.7131 per 100,000 population, surpassing the national average(0.125/100,000) [19]. As of 2023, a total of 1422 online-reported cases have been documented in Anhui Province during this year alone, far exceeding the annual count of last year(840 cases). This may be attributed to several factors. First, the continuous improvement of ecological conditions within Anhui Province over recent years has created a more favorable environment for natural tick vectors carrying SFTSV. Second, advancements in diagnostic capabilities and detection technologies have deepened our understanding of SFTS and enabled diagnosis for previously undiagnosed patients. Then, high incidence of patients is associated with an augmented risk of tick exposure due to increased outdoor activities among individuals in recent years [20]. The case fatality rate was 3.2% simultaneously, potentially underestimating the true value due to the absence of any subsequent follow-up of discharged patients [5]. While studies conducted elsewhere indicate that there is a distinct seasonal pattern and regional clustering trend associated with SFTS incidence [12–14], no relevant research has systematically described the spatio-temporal distribution characteristics specific to our province. Although the preliminary incidence data show that the situation of SFTS epidemic in our province is relatively serious, we do not know the further high-incidence areas and deep-seated causes, especially in recent years. Therefore, based on analyzing the incidence rates and epidemiological features across various counties and districts within our province, this study aims to provide an exploratory investigation into both temporal and spatial distribution features related to SFTS incidence.

Currently, the reported cases of SFTS in our province are primarily concentrated in Chuzhou City, Lu’an City, Hefei City, and Anqing City. The annual number of SFTS cases in these cities exhibits an increasing trend in recent years and shows signs of spreading to other regions such as Xuancheng City, Huangshan City, and Chizhou City. Global spatial autocorrelation analysis revealed significant clustering patterns for SFTS incidence across Anhui province from 2011 to 2023. Local spatial autocorrelation results indicate that high-high clustering and low-low clustering are the predominant patterns observed. The high-high concentration areas include Lai’an County, Langya District, Nanqiao District, Quanjiao County, Qianshan City, Lujiang County, Yuexi County, Shucheng County, and Huoshan County. Conversely, the low-low accumulation area comprises Xiao County, Lieshan District, Yongqiao District, Suixi County, Huaiyuan County, Huaishang District, Yingquan District, Lixin County, Guzhen County, and Panji District. During the period from 2011 to 2023, the aggregate area affected by SFTS in Anhui Province gradually increased. Anhui Province is situated in East China between 114°54 ‘-119°37’ east longitude and 29°41 ‘-34°38’ north latitude. It is bordered by the Yangtze River to the south and the Huai River to the north, dividing the province into three natural regions. The first region encompasses the plain area north of the Huai River. The second region lies between the Huai River and Yangtze River, characterized by rolling hills, rivers, and lakes. The final region is located in southern Anhui with its undulating mountains [21]. Spatial autocorrelation results demonstrated that low-low clustering area is predominantly situated in the plain region north of the Huai River, which is likely attributed to flat geography throughout these regions resulting in limited contact between local residents and ticks. whereas the high-high clustering area primarily exists in the southern regions of the Huai River.

In China, *H. longicornis* is the main transmission medium of SFTS. The researchers summarized and analyzed the geographical distribution, host diversity and specificity of ticks in China, anhui is one of the main geographical distribution areas of *H. longicornis* [22]. Futhermore, A preliminary identification of ticks in Anqing and Chuzhou(part of the main cluster areas) was conducted in the early stage of our research group, most of the adult ticks in parasitic ticks and free ticks in these two areas were identified as *H. longicornis* [23]. Meanwhile, an epidemiological survey indicate latent infection rate in healthy residents was 6.75% in 2016 and 10.91% in 2021 in Hefei City [20]. However, the current SFTS monitoring of population, host and ticks in our province is not perfect. In addition, previous study have found that direct contact with the blood or blood secretions of SFTS patients can also lead to SFTSV infection, and epidemiological investigations have shown that asymptomatic infection may be related to close contact with body fluids and mucous membranes of secondary patients [24]. All of these points to the possibility of human-to-human transmission of the virus. Moreover, in recent years, several studies have reported that patients are infected with SFTS through host animals (camels and pet dogs) [25, 26]. The transmission chain of SFTS is complex and there may be multiple modes of transmission. In the next step of our work, we need to carry out longitudinal follow-up investigation on the transmission rate of the population, host animals and vectors in various regions of Anhui Province, so as to grasp the epidemic trend and clarify the mode of communication of our province.

In terms of seasonal characteristics, the incidence of SFTS in Anhui Province reaches its peak mainly during April-May with a smaller peak observed in October. Generally, the number of haemaphysalis longicornata peaks twice in spring and autumn, and the number of the main peak tick in spring far exceeds the number of the secondary peak tick in autumn. Its epidemic time is from March to November every year, and the high incidence period is mostly from May to June [27, 28], which is consistent with the seasonal characteristics of SFTS incidence in our province.especially the seasonal fluctuation of ticks and human activities [29]. Similarly, environmental variables in different seasons also affect the occurrence of SFTS cases. Researchers used MaxEnt to explore the relationship between the distribution of SFTS cases and environmental variables, and found that factors such as altitude, yearly average temperature, yearly accumulated precipitation, and yearly average relative humidity would affect the occurrence of SFTS to different degrees [30]. Various evidence have shown that differences in environmental factors in different seasons may affect tick growth and the incidence of SFTS, and exert an influence on the months when it is most prevalent.

At present, the incidence of SFTS many be affected by various factors. SFTS is a natural epidemic disease carried by ticks and its spread and prevalence are inevitably affected by tick density distribution. Therefore, all factors affecting the survival, development, reproduction, behavior of the vector ticks can directly or indirectly affect the prevalence of SFTS, which remind us the necessity of step up our advocacy efforts. For example, temperature and precipitation can affect the occurrence of SFTS by directly changing the life cycle and spatial distribution of ticks [31, 32]. Within the appropriate temperature range, higher temperatures can shorten tick development and growth periods and increase egg laying and hatching rates [33]. Changes in precipitation may directly affect the ecology of tick breeding grounds. Previous studies on influencing factors of SFTS distribution found that meteorological factors(temperature, precipitation, etc.), microenvironmental conditions(percentage coverage of water bodies, forest and grassland coverage rate, etc.) and geographical topographic factor(altitude) had significant effects on tick survival and SFTS transmission. In addition, social factors such as economic level and Mammal density may also play a role in transmission [30, 32, 34]. In the future, we need to adopt a variety of methods such as model fitting analysis and epidemiological studies to carry out further analysis and research on relevant influencing factors.

Through spatio-temporal scanning analysis, we identified three main clusters of SFTS in Anhui Province in recent years: the first cluster encompasses several counties and districts within Chuzhou City, Ma’anshan City, and Hefei City, and is located in the lower reaches of the Yangtze River, the eastern region of Anhui Province; the second cluster primarily focused on the region of the Dabie Mountain range, and includes certain counties and districts within Lu’an City, Anqing City, and Hefei City; while the third cluster primarily focused on the region of the Huang Mountain range, and mainly comprises some counties and districts under Huangshan City, Xuancheng City Chizhou City, and Tongling City. Our analysis revealed that these SFTS cluster areas predominantly consist of hilly and mountainous terrains with abundant vegetation coverage, providing favorable conditions for vector tick survival. Notably, both Chuzhou and Ma’anshan are situated on the border between Anhui Province and Jiangsu Province which are two provinces with high SFTS incidence rates. Previous studies have also indicated that Xuyi County is location in Huai’an City(Jiangsu Province), which borders Chuzhou as another SFTS cluster area [13]. It is worth noting that in recent years, new areas with high incidence of SFTS have emerged successively in Anhui Province, such as some districts and counties in Hefei City, Huangshan City, and Xuancheng City. Moreover, recent study have shown that the number of SFTS cases in Hefei City has gradually increased in recent years, and the incidence area has the tendency that gradually shift from rural areas to urban streets [20]. Moreover, Lu’an City and Anqing City are located within the Dabie Mountain region, and Huangshan is a mountainous area where tea picking is a common livelihood activity among residents which may increase their exposure to transmission vector ticks potentially leading to more local SFTS cases [35]. Furthermore, Huangshan shares its border with Lin’an and Chun’an in Zhejiang Province—both considered as SFTS cluster areas [12]. We observed a strong concurrence between the clustering regions identified through spatiotemporal scanning and the high-high clustering regions detected by local spatial autocorrelation analysis. Furthermore, it is noteworthy that these clusters encompass multiple counties and districts, implying that future preventive measures against SFTS should involve collaborative prevention and control efforts across various regions to identify commonalities for effective management.

Our study also has corresponding limitations. Firstly, the incidence of SFTS and its vectors are evidently influenced by environmental factors. There also exist variations among counties and regions in terms of economic development, changes in diagnosis and treatment of SFTS, prevention and control measures for SFTS, as well as other factors. Environmental factors and other relevant variables were not incorporated into our analysis in this study; hence further investigation may be warranted in the future. Secondly, the case information included in this study was obtained from the infectious disease network reporting system; however, it is possible that the reported incidence of the disease does not entirely align with the actual situation because it is not a notifiable infectious disease. Our findings indicate a rising trend in annual SFTS incidence within Anhui Province over recent years, displaying evident seasonality patterns. Three cluster areas of SFTS were identified within Anhui Province: South of Huaihe River in eastern Anhui Province(including Chuzhou City and Maanshan City), Dabie Mountain cluster(mainly Lu’an City, Hefei City and Anqing City), as well as Huangshan cluster(mainly including Huangshan City, XuanCheng city and Tongling City). Future efforts towards prevention and control strategies for SFTS should prioritize these areas and identify the focus of disease vector control and monitoring prevention and control.

## Conclusion

We analyze the spatial autocorrelation and spatiotemporal clustering characteristics of SFTS in Anhui Province from 2011 to 2023. The incidence of SFTS in Anhui Province in 2011–2023 was spatially clustered. The spatiotemporal scanning results show three main clusters of SFTS in recent years.

## Data Availability

The data only can be acquired under the consent of the Anhui Center for Disease Control and Prevention.

## Abbreviations

SFTS: Severe fever with thrombocytopenia syndrome
SFTSV: Severe fever with thrombocytopenia syndrome virus
LISA: Local Indicators of Spatial Association

## References

[CR1] 1. Yu XJ, Liang MF, Zhang SY, et al. Fever with thrombocytopenia associated with a novel bunyavirus in China[J]. N Engl J Med, 2011,364(16):1523–1532. doi:10.1056/NEJMoa1010095 10.1056/NEJMoa1010095 PMC3113718 21410387

[CR2] 2. Tran XC, Yun Y, Van An L, et al. Endemic Severe Fever with Thrombocytopenia Syndrome, Vietnam[J]. Emerg Infect Dis, 2019,25(5):1029–1031. doi:10.3201/eid2505.181463 10.3201/eid2505.181463 PMC6478219 31002059

[CR3] 3. Lin TL, Ou SC, Maeda K, et al. The first discovery of severe fever with thrombocytopenia syndrome virus in Taiwan[J]. Emerg Microbes Infect, 2020,9(1):148–151. Published 2020 Jan 10. doi:10.1080/22221751.2019.1710436 10.1080/22221751.2019.1710436 PMC6968498 31918622

[CR4] 4. Li J, Li S, Yang L, Cao P, Lu J. Severe fever with thrombocytopenia syndrome virus: a highly lethal bunyavirus[J]. Crit Rev Microbiol, 2021,47(1):112–125. doi:10.1080/1040841X.2020.1847037 10.1080/1040841X.2020.1847037 33245676

[CR5] 5. Li H, Lu QB, Xing B, et al. Epidemiological and clinical features of laboratory-diagnosed severe fever with thrombocytopenia syndrome in China, 2011-17: a prospective observational study. Lancet Infect Dis. 2018;18(10):1127–1137. doi:10.1016/S1473-3099(18)30293-7 10.1016/S1473-3099(18)30293-7 30054190

[CR6] 6. Wang GS, Wang JB, Tian FL, et al. Severe Fever with Thrombocytopenia Syndrome Virus Infection in Minks in China[J]. Vector Borne Zoonotic Dis, 2017,17(8):596–598. doi:10.1089/vbz.2017.2115 10.1089/vbz.2017.2115 28654374

[CR7] 7. Liu K, Zhou H, Sun RX, et al. A national assessment of the epidemiology of severe fever with thrombocytopenia syndrome, China[J]. Sci Rep, 2015,5:9679. Published 2015 Apr 23. doi:10.1038/srep09679 10.1038/srep09679 PMC4407178 25902910

[CR8] 8. Miao D, Liu MJ, Wang YX, et al. Epidemiology and Ecology of Severe Fever With Thrombocytopenia Syndrome in China, 2010‒2018[J]. Clin Infect Dis, 2021,73(11):e3851-e3858. doi:10.1093/cid/ciaa1561 10.1093/cid/ciaa1561 PMC8664468 33068430

[CR9] 9. Estrada-Peña A, Ayllón N, de la Fuente J. Impact of climate trends on tick-borne pathogen transmission[J]. Front Physiol, 2012,3:64. Published 2012 Mar 27. doi:10.3389/fphys.2012.00064 10.3389/fphys.2012.00064 PMC3313475 22470348

[CR10] 10. Wu XB, Na RH, Wei SS, Zhu JS, Peng HJ. Distribution of tick-borne diseases in China[J]. Parasit Vectors, 2013,6:119. Published 2013 Apr 23. doi:10.1186/1756-3305-6-119 10.1186/1756-3305-6-119 PMC3640964 23617899

[CR11] 11. Yoshikawa T, Shimojima M, Fukushi S, et al. Phylogenetic and Geographic Relationships of Severe Fever With Thrombocytopenia Syndrome Virus in China, South Korea, and Japan[J]. J Infect Dis, 2015,212(6):889–898. doi:10.1093/infdis/jiv144 10.1093/infdis/jiv144 25762790

[CR12] 12. Tao M, Liu Y, Ling F, et al. Severe Fever With Thrombocytopenia Syndrome in Southeastern China, 2011–2019[J]. Front Public Health, 2022,9:803660. Published 2022 Feb 9. doi:10.3389/fpubh.2021.803660 10.3389/fpubh.2021.803660 PMC8864090 35223761

[CR13] 13. Liang S, Li Z, Zhang N, et al. Epidemiological and spatiotemporal analysis of severe fever with thrombocytopenia syndrome in Eastern China, 2011–2021[J]. BMC Public Health, 2023,23(1):508. Published 2023 Mar 16. doi:10.1186/s12889-023-15379-3 10.1186/s12889-023-15379-3 PMC10019416 36927782

[CR14] 14. Wang Y, Pang B, Ma W, et al. Spatiotemporal analysis of severe fever with thrombocytopenia syndrome in Shandong Province China, 2014–2018[J]. BMC Public Health, 2022,22(1):1998. Published 2022 Nov 1. doi:10.1186/s12889-022-14373-5 10.1186/s12889-022-14373-5 PMC9624039 36319995

[CR15] 15. Moran PAP. The Interpretation of Statistical Maps. J R Stat Soc Series B (Methodological). 1948;10(2):243–51.

[CR16] 16. Kulldorff M, Heffernan R, Hartman J, Assunção R, Mostashari F. A space-time permutation scan statistic for disease outbreak detection. PLoS Med. 2005;2(3):e59. 10.1371/journal.pmed.0020059 PMC548793 15719066

[CR17] 17. Bell BS. Spatial analysis of disease–applications. Cancer Treat Res. 2002;113:151–182. doi:10.1007/978-1-4757-3571-0_8 10.1007/978-1-4757-3571-0_8 12613354

[CR18] 18. Zargar TI, Alam P, Khan AH, et al. Characterization of municipal solid waste: Measures towards management strategies using statistical analysis. J Environ Manage. 2023;342:118331. 10.1016/j.jenvman.2023.118331 37315466

[CR19] 19. Chen QL, Zhu MT, Chen N, et al. Epidemiological characteristics of severe fever with thtrombocytopenia syndrome in China, 2011–2021. Zhonghua Liu Xing Bing Xue Za Zhi. 2022;43(6):852–859. doi:10.3760/cma.j.cn112338-20220325-00228 10.3760/cma.j.cn112338-20220325-00228 35725341

[CR20] 20. Zhang Q, Liu W, Wang W, et al. Analysis of spatial-temporal distribution characteristics and natural infection status of SFTS cases in Hefei from 2015 to 2021. Environ Health Prev Med. 2023;28:70. doi:10.1265/ehpm.23-00149 10.1265/ehpm.23-00149 PMC10654213 37967947

[CR21] 21. Jia L, Sun J, Fu Y. Spatiotemporal variation and influencing factors of air pollution in Anhui Province. Heliyon. 2023;9(5):e15691. Published 2023 Apr 23. doi:10.1016/j.heliyon.2023.e15691 10.1016/j.heliyon.2023.e15691 PMC10189381 37205997

[CR22] 22. Zhang YK, Zhang XY, Liu JZ. Ticks (Acari: Ixodoidea) in China: Geographical distribution, host diversity, and specificity. Arch Insect Biochem Physiol. 2019;102(3):e21544. 10.1002/arch.21544 PMC6850514 30859631

[CR23] 23. Song DD, Gong L,Wu JB, et al. Tick distribution in some epidemic areas of fever with thrombocytopenia in Anhui Province[J]. Anhui J Prev Med, 2020,26(04):267–269 + 280.DOI:10.19837/j.cnki.ahyf.2020.04.006.

[CR24] 24. Huang D, Jiang Y, Liu X, et al. A Cluster of Symptomatic and Asymptomatic Infections of Severe Fever with Thrombocytopenia Syndrome Caused by Person-to-Person Transmission. Am J Trop Med Hyg. 2017;97(2):396–402. 10.4269/ajtmh.17-0059 PMC5544100 28722592

[CR25] 25. Sun Y, Zhang D, Liu H, et al. The first reported cases of severe fever with thrombocytopenia syndrome virus from domestic sick camel to humans in China. Emerg Microbes Infect. 2024;13(1):2309990. 10.1080/22221751.2024.2309990 PMC10860415 38269573

[CR26] 26. Oshima H, Okumura H, Maeda K, et al. A Patient with Severe Fever with Thrombocytopenia Syndrome (SFTS) Infected from a Sick Dog with SFTS Virus Infection. Jpn J Infect Dis. 2022;75(4):423–426. 10.7883/yoken.JJID.2021.796 35228501

[CR27] 27. Zhang X, Zhao C, Cheng C, et al. Rapid Spread of Severe Fever with Thrombocytopenia Syndrome Virus by Parthenogenetic Asian Longhorned Ticks. Emerg Infect Dis. 2022;28(2):363–372. doi:10.3201/eid2802.211532 10.3201/eid2802.211532 PMC8798674 35075994

[CR28] 28. Sato Y, Mekata H, Sudaryatma PE, et al. Isolation of Severe Fever with Thrombocytopenia Syndrome Virus from Various Tick Species in Area with Human Severe Fever with Thrombocytopenia Syndrome Cases. Vector Borne Zoonotic Dis. 2021;21(5):378–384. doi:10.1089/vbz.2020.2720 10.1089/vbz.2020.2720 33535015

[CR29] 29. Hu YY, Zhuang L, Liu K, et al. Role of three tick species in the maintenance and transmission of Severe Fever with Thrombocytopenia Syndrome Virus[J]. PLoS Negl Trop Dis. 2020,14(6):e0008368. Published 2020 Jun 10. doi:10.1371/journal.pntd.0008368 10.1371/journal.pntd.0008368 PMC7307786 32520966

[CR30] 30. Sun JM, Wu HX, Lu L, et al. Factors associated with spatial distribution of severe fever with thrombocytopenia syndrome. Sci Total Environ. 2021;750:141522. doi:10.1016/j.scitotenv.2020.141522 10.1016/j.scitotenv.2020.141522 32846249

[CR31] 31. Jore S, Vanwambeke SO, Viljugrein H, et al. Climate and environmental change drives Ixodes ricinus geographical expansion at the northern range margin. Parasit Vectors. 2014;7:11. 10.1186/1756-3305-7-11 PMC3895670 24401487

[CR32] 32. Miao D, Dai K, Zhao GP, et al. Mapping the global potential transmission hotspots for severe fever with thrombocytopenia syndrome by machine learning methods. Emerg Microbes Infect. 2020;9(1):817–826. 10.1080/22221751.2020.1748521 PMC7241453 32212956

[CR33] 33. Yano Y, Shiraishi S, Uchida TA. Effects of temperature on development and growth in the tick, Haemaphysalis longicornis. Exp Appl Acarol. 1987;3(1):73–78. 10.1007/BF01200415 3453334

[CR34] 34. Jiang X, Wang Y, Zhang X, et al. Factors Associated With Severe Fever With Thrombocytopenia Syndrome in Endemic Areas of China. Front Public Health. 2022;10:844220. Published 2022 Feb 24. doi:10.3389/fpubh.2022.844220 10.3389/fpubh.2022.844220 PMC8907623 35284401

[CR35] 35. Gong L, Zhang Y, Wang J, et al. Evaluation of a community intervention program on knowledge, attitudes, and practices regarding severe fever with thrombocytopenia syndrome in Anhui Province, China. Front Public Health. 2022;10:891700. Published 2022 Oct 31. doi:10.3389/fpubh.2022.891700 10.3389/fpubh.2022.891700 PMC9659599 36388366

